# The Effect of Hybridisation on Mechanical Properties and Water Absorption Behaviour of Woven Jute/Ramie Reinforced Epoxy Composites

**DOI:** 10.3390/polym13172964

**Published:** 2021-08-31

**Authors:** Cionita Tezara, Agung Efriyo Hadi, Januar Parlaungan Siregar, Zalinawati Muhamad, Mohammad Hazim Mohamad Hamdan, Ahmed Nurye Oumer, Jamiluddin Jaafar, Agustinus Purna Irawan, Teuku Rihayat, Deni Fajar Fitriyana

**Affiliations:** 1 Department of Mechanical Engineering, Faculty of Engineering and Quantity Surveying, INTI International University, Nilai 71800, Malaysia; 2 Mechanical Engineering Department, Faculty of Engineering, Universitas Malahayati, Jl. Pramuka No. 27, Kemiling, Bandar Lampung 35153, Indonesia; 3 College of Engineering, Universiti Malaysia Pahang, Gambang 26300, Malaysia; nurye@ump.edu.my (A.N.O.); jamiluddin@ump.edu.my (J.J.); 4 Department of Mechanical Engineering, Politeknik Sultan Haji Ahmad Shah, Semambu, Kuantan 25350, Malaysia; zalina4359@gmail.com; 5 Faculty of Engineering and Computing, First City University College, No. 1, Persiaran Bukit Utama, Bandar Utama, Petaling Jaya 47600, Malaysia; 6 Faculty of Engineering, Universitas Tarumanagara, Jakarta 11480, Indonesia; agustinus@untar.ac.id; 7 Department of Chemical Engineering, Politeknik Negeri Lhokseumawe, Lhokseumawe 24301, Indonesia; teukurihayat@yahoo.com; 8 Department of Mechanical Engineering, Universitas Negeri Semarang, Kampus Sekaran, Gunungpati, Semarang 50229, Indonesia; deniifa89@mail.unnes.ac.id

**Keywords:** jute fibre, ramie fibre, mechanical properties, hybrid, natural fibre, water absorption

## Abstract

Recently, the most critical issue related to the use of natural fibre-reinforced polymer composites (NFRPC) is the degradation properties of composites exposed to the environment. NFRPC’s moisture absorption behaviour has adverse effects on the composite’s mechanical properties and dimensional stability. The purpose of this study is to analyse the mechanical properties of epoxy composites reinforced by jute–ramie hybridisation. This study also analysed the effect of stacking sequence hybridisation of the jute–ramie composite on water absorption behaviour. A five-layer different type of stacking sequence of single and hybrid jute–ramie is produced with the hand lay-up method. The results obtained from this study found that the mechanical properties and water absorption behaviour of a single jute fibre are lower compared to a single ramie fibre. The hybrid of jute–ramie has been able to increase the performance of composite compared to pure jute composites. The mechanical properties of the hybrid jute–ramie composite show a reduction effect after exposure to an aqueous environment due to the breakdown of fibre matrix interfacial bonding. However, after 28 days of immersion, all types of the stacking sequence’s mechanical properties are still higher than that of pure epoxy resin. In conclusion, the appropriate sequence of stacking and selecting the material used are two factors that predominantly affect the mechanical properties and water absorption behaviour. The hybrid composites with the desired and preferable properties can be manufactured using a hand-lay-up technique and used in the various industrial applications.

## 1. Introduction

One common drawback of natural fibre-reinforced polymer composites in outdoor applications is their moisture absorption behaviour, which affects the dimensional stability and mechanical properties of the composites [1,2]. Numerous studies have been done to address this issue [3,4,5,6]. However, the absorption of moisture from natural fibre composites remains a major concern, especially for outdoor applications [7,8].

As previously mentioned, the water absorption behaviours in natural fibre are the well-known limitation for implementing natural fibre as a reinforcement agent for polymer composites [9]. Most previous studies reported that the fibre composite’s tensile strength and flexure strength are significantly reduced after water immersion [2,10,11,12]. Water absorption in fibre-reinforced polymer composites is mediated by three major mechanisms: Diffusion, capillary, and water molecule transport. The diffusion mechanism takes place between the micro gaps in the polymer chains. Certainly, water diffusion at the fibre–matrix interface causes differential swelling of the natural fibre, which is mostly due to its hydrophilic nature. As a result, fibre swelling may induce tension at the interface, resulting in matrix degradation and micro-cracking, which exacerbates water uptake [13]. Capillary transport occurs in the gaps at the fibre–matrix interface space if the reinforcement was not completely impregnated with the matrix during the manufacturing process [7]. The transport of water molecules through microcracks that can occur in the matrix as a result of fibre swelling is especially relevant in natural fibre composites [14]. In this light, the moisture diffusion in NFRPC material depends on a specific factor or parameter that involves the volume fraction of fibre, the fibre sequence, fibre treatment, the viscosity of the matrix, voids, temperature, and humidity [15,16,17,18,19].

The polymer composite’s fibre content can generally affect water absorption and mechanical properties before and after moisture absorption [20]. Numerous studies related to the composite’s fibre content have been carried out in recent years [21,22,23,24]. The immersion of the natural cellulosic fibres reinforced composites in water with different concentrations of fibre content. Furthermore, the result shows that the absorption process’s fibre content has affected the composites’ water absorption [16,25]. Water absorption behaviours, such as water gain and thickness swelling, increase with the increasing fibre content or fibre layering. However, the tensile strength and tensile modulus of the composite decreased after being immersed in water for a particular time [7]. Furthermore, polymer composites are subject to degradation by environmental chemical, physical, and biological stresses [26]. Moisture, temperature, pH, salinity, prolonged pressures, and microorganisms are all important environmental elements that might affect the durability of polymer composites. In most marine, terrestrial, and coastal areas where polymer composites are likely to be used, microbial activity is an essential environmental aspect. Microbial interactions with polymer composites should be calculated in future modelling of polymer composites designed for long-term durability and lifetime. A higher loss in molecular weight caused by the exposure period to microorganisms increased. The microbial activity drives polymer chain scission-based degradation of polymer composites, resulting in a significant decrease in the starting melting temperature. Polymer degradation caused by microbial activity results in a decrease in modulus and hardness as well as an increase in displacement [26,27].

Physical and chemical treatment of fibres is a potential solution to the high moisture absorption problem. Another alternative solution is to use hybrid composites, which can overcome some of the natural fibre composites drawbacks thereby allowing for the customization of their properties. The hybrid method can be used to create low-cost composites while maintaining the quality of mechanical and thermal properties. Furthermore, hybrid composites have balanced mechanical strength, which single-fibre reinforced composites cannot produce [28]. The hybridisation of the natural laminated fibre in the composite is one parameter that can affect the result of water absorption by the composites [14,29]. A previous study investigated the water absorption of hybrid composites made from oil palm empty fruit bunches with jute fibres. It was found that the hybrid composites of oil palm empty fruit brunch and jute fibre have better water resistance and dimensional stability than the pure oil palm empty fruit brunch fibre composite. The pure composite showed 21.39% water absorption, while the hybrid fibre composite exhibited the highest water absorption of 11.20% [30]. A similar result was found by Saw et al. [31], reporting that a pure coir composite has higher water absorption with 19.74% than the hybrid composite of coir and jute fibre at 15.3%. Other studies [32,33] reported that a sisal/roselle hybrid composite showed a great reduction in tensile and flexure strength after water immersion. This is due to the water invasion, which weakened the adhesion bond between the fibre and matrix.

Hence, it is crucial to study the moisture absorption behaviour of hybrid natural fibres in natural fibre-reinforced composites to predict the possible consequences caused by water absorption. This study investigates the effect of tensile and water absorption properties of the hybridisation of jute and ramie as the reinforcement of epoxy resin.

## 2. Materials and Methods

### 2.1. Materials

**Figure 1:**
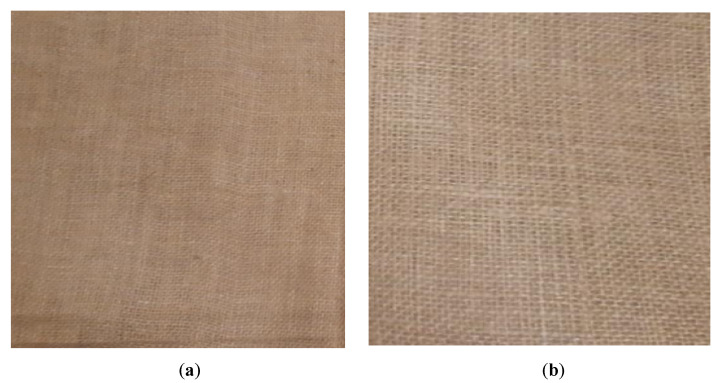
Image of (**a**) plain-woven ramie (**b**) plain-woven jute.

The plain-woven jute and the ramie fibre (commercial product) as reinforcement for epoxy resin were purchased from Impiana Enterprise located at Kuala Lumpur, Selangor (Figure 1). The fibres were cleared and washed thoroughly using mild conditions to remove any unwanted impurities. After that, the fibres were dried in an oven at around 80 °C for 24 h to remove excess moisture. Lastly, the fibres were cut into a suitable dimension of 30 cm (length) × 30 cm (width) to ensure they could perfectly fit into the mould. Epoxy resin (816A) with a density of 1.2 g/cm^3^ and hardener (651) were purchased from Southern Strait Engineering, Johor, Malaysia, and used as a matrix to produce the composite laminate.

### 2.2. Fabrication of Laminate Composites

**Figure 2:**
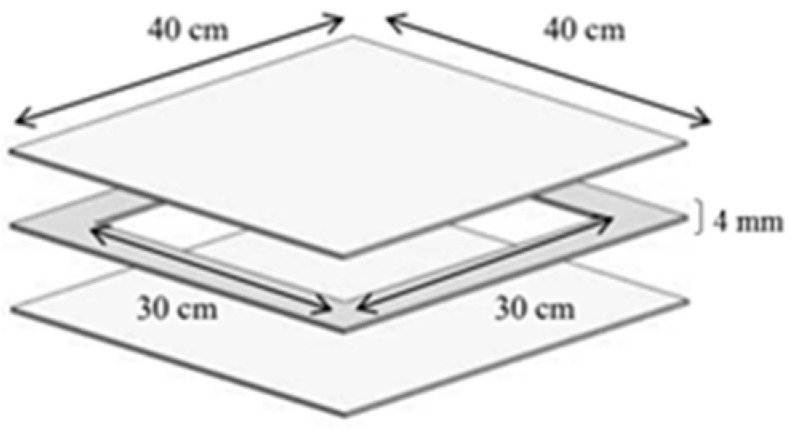
Moulding dimensions.

A suitable mould was prepared with the dimensions of 30 cm (L) × 30 cm (W) × 4 mm (T) to fabricate the composite plates by stacking the fibre layer by layer with the epoxy resin matrix as shown in Figure 2. The hand lay-up technique was utilised to produce a composite plate. The hand lay-up technique offers several advantages over other methods due to its simplicity and minimal reliability on machines [34].

**Figure 3:**
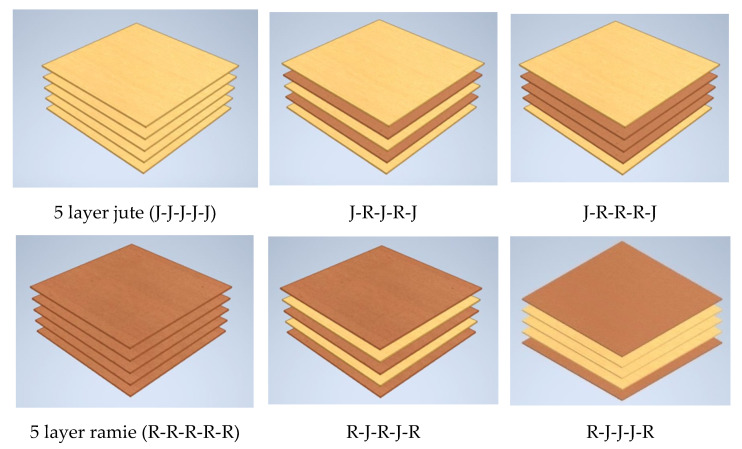
Schematic of stacking sequence of hybrid jute–ramie composites.

For this investigation, the 5-layer plain-woven arrangement of single jute, ramie, and hybrid jute–ramie reinforced epoxy composites are illustrated in Figure 3. First, a releasing agent was sprayed on the mould’s inner surfaces before the laminating process started, to prevent the composite from sticking to the mould’s surface and for ease of removal. The epoxy resin was then poured into the mould, followed by the placement of the woven fibres. The process was repeated until the 5 layers of woven jute, ramie, and hybrid jute–ramie of the composite plates were created. Next, the moulds underwent a curing process where they were left for roughly 24 h at room temperature to remove the air trapped inside the laminated composites and allow the resin to harden. Afterwards, the laminated fibre woven composites were cut according to the ASTM standard. A total of six (6) specimens were prepared and tested for various properties such as tensile, flexural, and water absorption tests.

### 2.3. Tensile Test

One of the most widely used mechanical tests for determining materials’ mechanical properties is the tensile test. Tensile testing helps determine some of the critical parameters, such as tensile strength and tensile modulus, that the material could withstand before it breaks. The tensile test was carried out on an INSTRON 3369 universal testing machine with a crosshead speed of 2 mm/min. In this study, the ASTM D638–IV standard for tensile testing, with its respective specimen dimension, was followed.

### 2.4. Flexural Test

Flexural specimens were prepared according to the requirements of ASTM D790. The most well-known bending test for composite materials is the 3-point bending test. The flexural test was performed using the INSTRON 3369 universal testing machine with a 2 mm/min crosshead speed. Then, the flexural strength and flexural modulus were recorded.

### 2.5. Water Absorption Test

The water absorption test was carried out within a four-week (1, 7, 14, 21, and 28 days) period to determine the percentage of weight gain due to the water immersion for various types of tensile specimens. The specimens’ weekly weight changes were measured using a high-precision weighing balance, which could provide results up to 4 decimal places.

### 2.6. Scanning Electron Microscopy (SEM)

Zeiss Evo50 Scanning Electron Microscopy (SEM) was used to analyse the tensile fracture of jute–ramie reinforced epoxy resin composites. It has a tungsten hairpin thermionic electron gun and can function from a low vacuum (5 Pa) to high chamber pressures of up to 3000 Pa in either a water vapor or nitrogen atmosphere (air). The secondary electron (SE) detector and the backscattered electron (BSE) detector are utilized with a working distance of 10 mm (at Analytical Working Distance) [35]. The specimens were sputter-coated with a thin layer of palladium and placed on the SEM holder through double-sided electrically conducting carbon adhesive tapes to avoid surface charge when exposed to the electron beam on the specimens. Finally, the samples were examined under a microscope using 15 kV of acceleration tension and magnification of ×200.

## 3. Result and Discussion

### 3.1. Tensile Properties

The investigation of the effect of the layering sequence on tensile properties of single jute, ramie, and hybrid composite that are performed in this study is based on the findings of a previous study by Dhakal et al. [19]. Concerning the effect of the layering sequence from two to five layers on the mechanical properties of a hemp-reinforced unsaturated polyester composite, it was observed that the five-layer hemp fibre achieved the highest mechanical properties (tensile and flexural properties) [19].

The response of tensile strength and tensile modulus to different layering sequences in hybrid jute–ramie reinforced epoxy was analysed using a one-way analysis of variance (ANOVA) as shown in Table 1 and Table 2. The tensile strength has a *p*-value of 0.001 and the tensile modulus has a value of 0.0000007. Both values obtained are less than the 0.05 level of significance. Thus, the null hypothesis, which stated that there was no relationship between the layering sequences of the hybrid jute–ramie reinforced epoxy, can be rejected.

**Figure 4:**
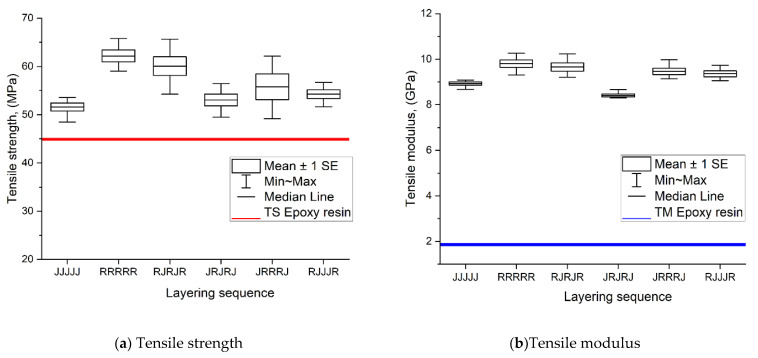
Tensile strength (**a**) and tensile modulus (**b**) of single and hybrid jute–ramie woven composites.

The tensile strength (TS) and tensile modulus (TM) of the five-layer single jute, ramie, and hybrid jute–ramie composites with different stacking sequences are presented in Figure 4. Previous studies found that the TS and TM of single jute fibre are in the range of 393–773 MPa and 26.5 GPa, which are lower than the ramie fibre of 400–938 MPa and 61.4–128 GPa, respectively [36,37]. In this study, the five-layer ramie composite registered TS and TM values of 62 MPa and 9.8 GPa, respectively (an increment of 34% and 427% for TS and TM, respectively, compared to epoxy resin). On the other hand, the five-layer jute composites showed values of 52 MPa and 8.9 GPa for TS and TM, respectively (14% and 380% increment compared to epoxy resin samples).

The stacking sequence that produced the highest strength with the fibre (Ramie) at the top (exterior) was R-J-R-J-R with 60 MPa and 9.66 GPa (34% and 419%), which is slightly higher than J-R-J-R-J with 53 MPa and 8.4 GPa (18% and 315%). Theoretically, the high strength and modulus fibre provided at the top and bottom layers withstood the applied load, whereas the core absorbed and distributed the loads uniformly [38]. These results are in line with another study stating that when the high tensile strength of ramie was placed at the top, middle (core), and bottom skin (R-J-R-J-R) of the composite, it provides high tensile properties compared to J-R-J-R-J. All the work shows that the high strength fibre used as the skin helps to enhance the TS and TM of the hybrid composite. For the other stacking sequence, J-R-R-R-J (56 MPa and 9.46 GPa) has slightly higher strength compared to R-J-J-J-R with 54 MPa and 9.36 GPa. In other words, having more ramie layers in the core leads to higher TS and TM compared to having two layers of ramie at the skin surface. The results of the hybridisation of the jute–ramie composite has brought improvement compared to the single jute composites. This finding is supported by several similar studies that found that the stacking sequence plays a major role in determining the mechanical properties of hybrid natural fibre-reinforced composites [39,40,41]. This finding can be used to conclude the result of the tensile properties in this study.

The present investigation was compared to previous research studies on hybrid composite reinforced epoxy. The majority of previous research has demonstrated the effect of hybridising glass and jute with a different layering sequence on the tensile properties. The results indicate that when compared to neat epoxy, the jute fibre and hybrid composite provide encouraging results. The tensile strength of the hybrid jute–ramie composite is almost identical to that of the four-layer glass–jute composite. Nonetheless, the hybrid jute–ramie fibre produces a higher tensile modulus than the glass–jute fibre. The comparison of jute–ramie reinforced epoxy composites to other types of hybrid composites is detailed in Table 3.

### 3.2. Flexural Properties

Table 4 and Table 5 show the ANOVA results for the flexural strength and the flexural modulus. The *p*-values for flexural strength and modulus are less than 0.05, indicating that they are statistically significant. There is a strong correlation between the flexural strength and flexural modulus of the hybrid jute–ramie reinforced epoxy and the layering sequence.

**Figure 5:**
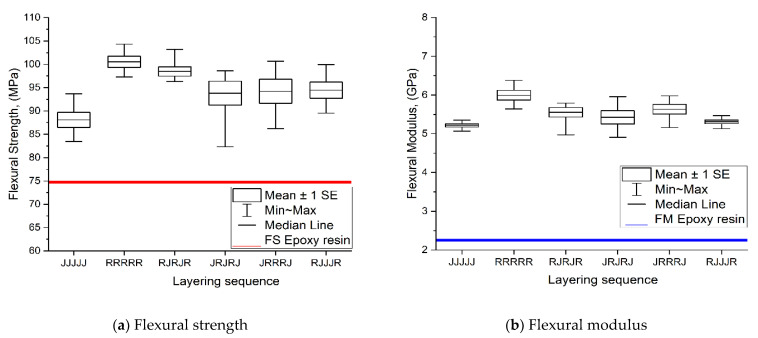
Flexural strength (**a**) and flexural modulus (**b**) of single and hybrid jute–ramie woven composites.

Figure 5 shows the flexural strength (FS) and flexural modulus (FM) of the composites with different jute, ramie, and hybrid stacking sequences. The graph shows a similar trend with the tensile properties, where the addition of single and hybrid jute–ramie significantly enhanced the FS and FM of the composites. The single five-layer ramie composite showed the highest FS and FM values of 100 MPa and 5.5 GPa, respectively. The increment brought by the single ramie in epoxy resin is about 34% for FS and 144% for FM. In comparison, the FS and FM of the jute fibre have shown only about 18% and 114% increments, respectively. Since the jute fibre has the lowest FS and FM, using different hybrid jute–ramie stacking sequences improves the flexural properties. Comparing the hybrid composites, R-J-R-J-R has higher FS and FM compared to J-R-J-R-J. Ramie has higher specific strength than the jute fibre and will be placed at the outer layer. The core layer then results in better flexural properties than when the jute fibre is placed at the outer and core layers. Several studies found that this mechanism also affects hybrid natural fibre composites [46,47,48]. Moreover, applying the proper hybrid jute–ramie stacking sequence affects the composites’ properties, as mentioned in the previous studies [49,50].

### 3.3. Water Absorption

**Figure 6:**
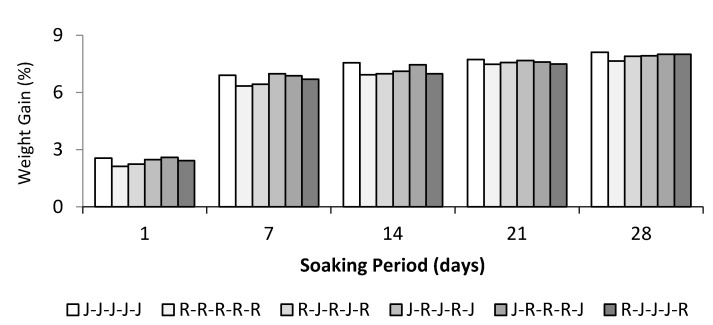
Water absorption of pure and hybrid jute–ramie composites.

Figure 6 illustrates the water absorption behaviours of five-layer single jute, ramie, and hybrid jute–ramie composites immersed in distilled water with different immersion times. The graph shows that the fibre’s weight increased proportionally with the immersion period. As can be seen from the graph, the maximum percentage weight gain of the five-layer single and hybrid composite tensile specimens after they were immersed in water for 28 days (672 h) registered the following results (in descending order): Jute (8.10%) > R-J-J-J-R (8.02%) > J-R-R-R-J (8.01%) > J-R-J-R-J (7.93%) > R-J-R-J-R (7.90%) > five-layer ramie (7.58%).

The results show that ramie fibre is more water resistant than jute fibre. After 24 h of immersion, the weight gained by the ramie fibre composite (2.13%) is less than the weight gained by the jute composite, which is 2.65%. Other studies investigated six and eight layers of a flax-reinforced bio-epoxy composite, where the weight gain by the tensile specimen after being immersed for 768 h is about 6.23% and 8.71%, respectively [51]. The hydrophilicity character of natural fibres implies that the fibre showed high moisture absorption, which could be due to the failure of certain parts of the manufactured composites under wet conditions. This is caused by the fibres’ swelling or delamination on the composites’ surface [49,52]. In general, the moisture diffusion in a composite depends on factors such as the volume fraction of fibre, voids, the viscosity of matrix, humidity, and temperature [25]. However, when the water absorption of the pure jute and hybrid jute–ramie composites are compared, an overall reduction of water absorption compared to the pure jute composite was observed. With hybridisation, the resistance to water absorption is greatly improved [2]. The water absorption graph is laid between the single jute composite’s water absorption curve for the hybrid jute–ramie composite. A similar result was also reported by Jawaid et al. (2011), who experimented on the hybrid composite made from oil palm empty fruit bunches/jute fibres [30]. Investigation of the tensile and flexural strength of hybrid composites made from the oil palm empty fruit bunches/jute fibres reinforced epoxy [30] was done and they concluded that the tensile and flexural properties of the hybrid composite were found to be higher than the oil palm empty fruit bunches composite but lower than those of the woven jute composite. The influence of the layering pattern on the water absorption and thickness swelling of hybrid composites was investigated by Khalil, H.P.S.A. et al. [53]. The woven fibre mats of jute (Jw) and oil palm empty fruit bunches (EFB) were utilized to make hybrid composites, which were subsequently impregnated with epoxy resin. They observed that EFB fibre composites showed the maximum water absorption during the complete duration of immersion. The hybridisation of EFB composites with woven jute fibre showed advantageous effects on the water absorption and thickness swelling by improving fibre/matrix bonding [53]. The higher water resistance of the hybrid composite than the single jute composite has also been proven [54]. Furthermore, because its epoxy resin matrix possesses water-resistant qualities, the use of epoxy resin in fibre–mat composites could prevent water absorption [53].

The current research’s flexural properties are also compared to those of a previous study in Table 6. The available data in the literature are typically for hybrid composites composed of natural and synthetic fibres. Hybrid composites reinforced with natural fibres, which are frequently combined with synthetic fibres such as glass fibres, can also exhibit excellent mechanical properties. According to Table 6, the flexural strength of jute–ramie hybrid reinforced epoxy is comparable to that of glass fibre-reinforced epoxy. Similarly, the modulus of elasticity is greater than that of other hybrid composite reinforced epoxy materials.

### 3.4. Thickness Swelling

**Figure 7:**
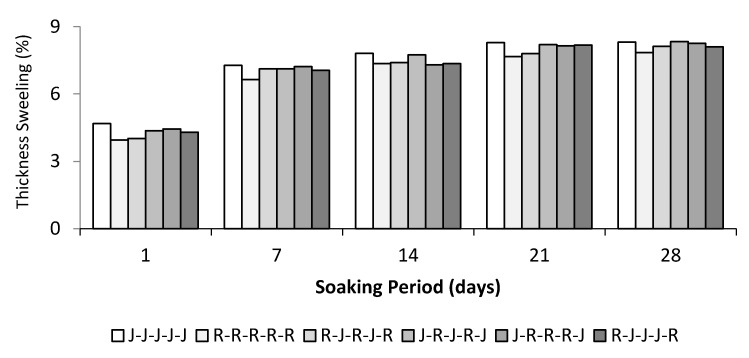
Thickness swelling of jute–ramie hybrid composites.

Theoretically, cellulosic fibres’ swelling causes stress in the interfacial regions, leading to the degradation of natural fibre composite properties. This causes micro-cracking in the matrix close to the swollen fibres, indicating transport and capillarity through the micro cracks. Figure 7 shows the function graph of thickness swelling of single jute, ramie, and hybrid jute–ramie composites immersed in distilled water under room temperature against the immersed time. Since water can act as a plasticizer, moisture absorption in natural fibre composites can affect dimensional stability and composites’ mechanical properties [13]. As observed from the graph, the specimens’ thickness swelling in different stacking sequence arrangements is proportional to the immersion time. This trend is analogous to a similar study on the effect of water absorption on natural fibre-reinforced polymer composites [56].

It was observed that at the beginning, the curve showed linear behaviour that started to slow down and eventually reached the saturation point after a certain period of immersion time [25]. It was shown that the thickness swelling of pure and hybrid jute–ramie composites increased significantly between day 1 and day 7 of immersion in distilled water. It was observed from the graph that the curves showed a rather steep trend between days 1 to 7 days of the immersion time. However, the graph flattens starting from 168 h onwards. The five-layer jute woven composites showed the highest thickness swelling (8.31%) compared to the five-layer ramie composite (7.84%) after immersion for 28 days. A similar study reviewed the equilibrium moisture content of jute fibre at 65% relative humidity (RH) and 21 °C and reported a thickness swelling of about 12%. Meanwhile, the ramie fibre showed a thickness swelling of only 9% [57]. Consequently, the properties of jute fibre are more hydrophilic compared to ramie fibre. When natural fibre composites are exposed to moisture, the water molecules will diffuse into the composite and bind to natural fibre hydrophilic groups, forming intermolecular hydrogen bonding with the fibres mitigating the interfacial adhesion of the interfacial fibre/matrix [15].

**Figure 8:**
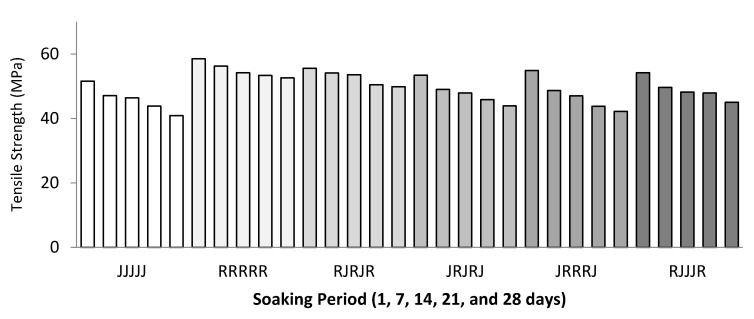
Water absorption behaviour on the tensile strength of single and hybrid composites.

**Figure 9:**
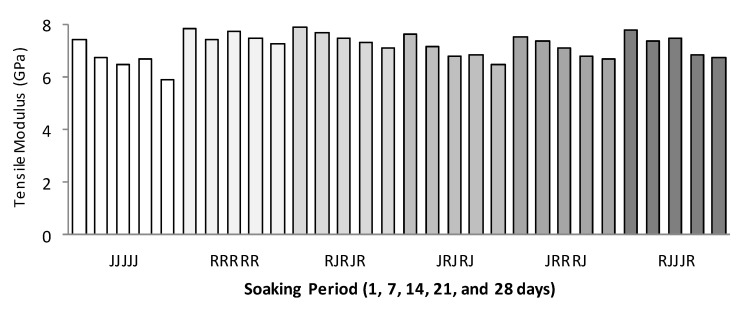
Water absorption behaviour on the tensile modulus of single and hybrid composites.

Figure 8 and Figure 9 show the effects of water absorption degradation on the TS and TM of jute for the ramie and hybrid composite after being immersed for 28 days. The graph’s trend shows that the TS and TM linearly decrease for all single and hybrid composites after being immersed for 24 h to 672 h.

The decrement of the strength can be attributed to an increase in water absorption percentage in different soaking times, while the thickness swelling is caused by the higher number of micro-cracks due to the thickness swelling [7,58,59]. Then, when the axial loads are applied, it weakens the fibre–matrix interface area.

**Figure 10:**
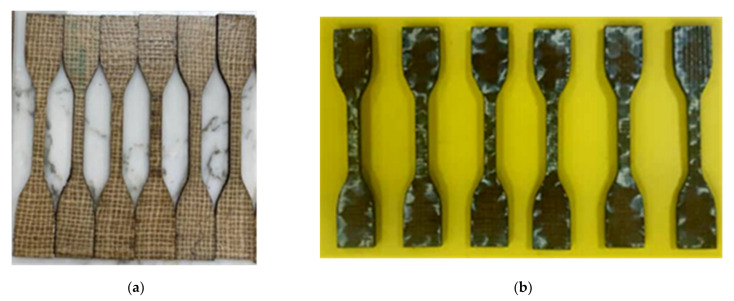
Tensile specimen (**a**) before and (**b**) after being immersed in distilled water for 28 days.

Figure 10a,b shows the tensile specimen before and after immersion. Figure 10b clearly shows the water molecule penetration/transport and capillarity through the micro-cracks, especially at the tensile specimen’s surface. From the observation, TS of the five-layer single jute fibre composites decreased by 21% from 51.60 MPa (without immersion) to 40.90 MPa (immersed for 28 days) while TM was reduced by 33% from 8.90 GPa to 6.51 Gpa.

Meanwhile, for ramie, the TS and TM decreased to 15% and 21%, respectively. The percentage reduction of jute fibre in TS and TM is relatively higher compared to ramie fibre. The presence of hydroxyl and other polar groups in jute fibre causes the composite to have strong hydrophilic properties [60], resulting in incompatibility and low wettability in the matrix of hydrophobic polymers, and subsequently weak interfacial properties [61]. The TS and TM of hybrid jute–ramie composites range 17–24% and 23–30%, respectively. This study concluded that the performance of hybrid jute–ramie reinforced epoxy enhances the composite’s water-resistance property, resulting in an improved tensile strength and tensile modulus compared to single jute fibre composites.

**Figure 11:**
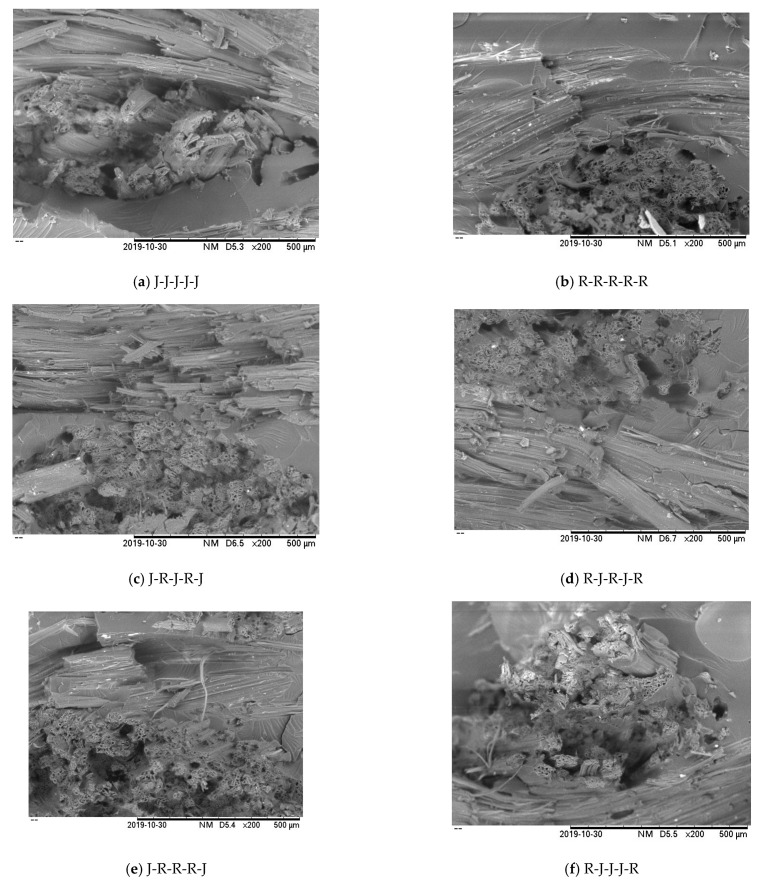
SEM analysis on the tensile surface fracture (magnification 200×) of single and hybrid jute–ramie composites (**a**–**f**).

Figure 11a–f shows the tensile fracture surfaces of single jute, ramie, and hybrid jute–ramie composites under scanning electron microscopy (SEM). Researchers normally use the SEM micrograph to investigate the tensile properties of composites’ correlation and the specimen’s fracture surface. Fibre pull-out, matrix crack, void, and debonding are significant factors that influence the interfacial adhesion between the fibre and the matrix [61,62].

Figure 11a illustrates that for the single five-layer jute composite, with magnification of 200×, the fibre pull-out and debonding has appeared on the specimen’s surface. Fibre pull-out itself is one of the failure mechanisms in NFRPC under tensile test [63]. In the meantime, debonding refers to the fibre’s embedded quality in the matrix [64]. The micrograph has proven weak interfacial adhesion between jute fibre and epoxy resin in tensile strength (52 MPa). Similar results observed by Ahmed et al. (2007) indicated that the woven jute laminated polyester composites performed worse in terms of damage resistance and tolerance than jute/glass hybrid laminates [65]. Figure 11b shows that the micrograph of single ramie has less fibre pull-out than jute fibre, leading to a good interfacial bond between ramie fibre and epoxy resin, resulting in a higher tensile strength (62 MPa) compared to other types of composites. The micrographs of the hybrid jute–ramie are shown in Figure 11c–f.

When the ramie fibre is placed at the outer layer (skin) of the hybrid, for example, R-J-R-J-R and R-J-J-J-R, it has shown lower fibre pull-out compared to J-R-J-R-J and J-R-R-R-J. Nevertheless, all hybrid stacking sequence combinations in this study have enhanced the tensile properties compared to the single jute fibre. The use of natural fibre hybrid composites has garnered many researchers’ interest due to their ability to increase the composites’ mechanical properties [2,48,66].

The findings of this study agree with previous researchers’ arguments that the mechanical and physical properties of natural fibre woven reinforced polymer thermosetting composites are affected by several factors, including (i) composite parameters such as fibre source, reinforcement types, laminate sequence, reinforcement quantities, the content of woven fibre in polymer, the orientation of the fibre, and the layering sequence of woven fibre, and (ii) fabrication process parameters, which include the processing techniques, processing temperature, pressure level, etc. [67,68,69].

## 4. Conclusions

This study has successfully investigated the effect of hybridization of jute–ramie reinforced epoxy composites on mechanical properties and water absorption behaviour. The results showed that the mechanical properties and water absorption behaviour of hybrid jute–ramie, specifically, the TS, TM, weight gain, thickness swelling, and degradation of tensile properties, were improved after the specimen was immersed in water for 28 days. Jute fibre has lower mechanical properties, hence the hybridisation of such fibres with other fibres that have higher mechanical properties (such as ramie fibre) is one of the alternatives to enhance the performance of the jute fibre that could produce a better outcome in comparison with the method of modifying the natural fibre with a chemical treatment coupling agent. The sequence of R-J-R-J-R has demonstrated the maximum mechanical strength compared to the other hybrid composites. The proper stacking sequence is the crucial factor to determine the quality of hybrid composites materials. The use of hybrid natural fibres in this study has shown satisfactory results. However, the use of natural fibre woven reinforced polymer composites is not recommended for outdoor applications because the tensile strength of the composites is decreased when being exposed to water. As a suggestion from the results of this study, the hybrid composite could be used for non-structural and industrial applications, such as manufacturing indoor parts in the automotive and furniture industry.

## Figures and Tables

**Table 1:** ANOVA of the tensile strength of the hybrid jute–ramie reinforced epoxy.

Source	DF	Adj SS	Adj MS	F-Value	*p*-Value
Factor	5	428.9	85.78	6.65	0.001
Error	24	309.4	12.89		
Total	29	738.3			

**Table 2:** ANOVA of the tensile modulus of the hybrid jute–ramie reinforced epoxy.

Source	DF	Adj SS	Adj MS	F-Value	*p*-Value
Factor	5	6.753	1.35054	15.63	0.0000007
Error	24	2.073	0.08638		
Total	29	8.826			

**Table 3:** Comparison of tensile properties of the hybrid reinforced epoxy composite.

Type of Fibre Hybrid	Type of Reinforcement	Fibre Arrangement and Layering Size	Percentage Fibre Content (%)	Tensile Strength		Reference
				Tensile Strength (MPa)	Tensile Modulus (GPa)	
Ramie/Jute	Bi-directional	J-J-J-J-J (5)		51.6	8.93	Current study
		R-J-R-J-R (5)		60	9.66	
		J-R-J-R-J (5)		53.1	8.40	
		J-R-R-R-J (5)		55.8	9.46	
		R-J-J-J-R (5)		54.3	9.36	
Glass fibre/Jute	Bi-directional	J-J-J-J (4)	18.5	52	2	[42]
		G-J-G-J (4)	17.5	78	3	
		J-G-G-J (4)	17.5	74	2.6	
		G-J-J-G (4)	17.5	88	4.8	
Glass fibre/Jute	Bi-directional	G-J-J-J-G (1)	-	46.5	2.5	[43]
E-glass fibre/Jute	Bi-directional	J-J-G-G-G-J-J (7)		50	-	[44]
		J-J-J-J-J-J (6)		84	-	
		G-G-J-J-J-J-G-G (8)		125	-	
Carbon fibre/Jute	Bi-directional	C-C-J-C-C (5)	42	257.6	9.8	[45]
		C-J-C-J-C (5)	39.1	172.8	7.9	
		C-J-J-J-C (5)	32	108.3	5.7	

**Table 4:** ANOVA of the flexural strength hybrid jute–ramie reinforced epoxy.

Source	DF	Adj SS	Adj MS	F-Value	*p*-Value
Factor	5	557.1	111.42	5.22	0.001
Error	30	640.2	21.34		
Total	35	1197.3			

**Table 5:** ANOVA of the flexural modulus hybrid jute–ramie reinforced epoxy.

Source	DF	Adj SS	Adj MS	F-Value	*p*-Value
Factor	5	2.320	0.46409	5.79	0.001
Error	30	2.404	0.08013		
Total	35	4.724			

**Table 6:** Comparison of flexural properties of hybrid composite reinforced epoxy.

Type of Fibre Hybrid	Type of Reinforcement	Fibre Arrangement and Layering Size	Percentage Fibre Content (%)	Flexural Strength		Reference
				Flexural Strength (MPa)	Flexural Modulus (GPa)	
Ramie/Jute	Bi-directional	J-J-J-J-J (5)		88.1	5.22	Current study
		R-J-R-J-R (5)		98.4	5.55	
		J-R-J-R-J (5)		93.8	5.42	
		J-R-R-R-J (5)		94.45	5.31	
		R-J-J-J-R (5)		94.22	5.63	
Glass fibre/Jute	Bi-directional	J-J-J-J (4)	18.5	72	3.4	[42]
		G-J-G-J (4)	17.5	164	6.6	
		J-G-G-J (4)	17.5	96	4.6	
		G-J-J-G (4)	17.5	132	5.4	
Glass fibre/Jute	Bi-directional	G-J-J-J-G (1)		11.9	1.21	[43]
E-glass fibre/Jute	Bi-directional	J-J-G-G-G-J-J (7)		7	-	[44]
		J-J-J-J-J-J (6)		6	-	
		G-G-J-J-J-J-G-G (8)		11	-	
Oil palm empty fruit bunches/Jute	Bi-directional	EFB-J-EFB (3)		44.3	2.68	[55]
		J-EFB-J (3)		49	3.07	

## Data Availability

Data are contained within the article.
